# Supplementary material for: TOR and heat shock response pathways regulate peroxisome biogenesis during proteotoxic stress
Source: Nat Commun. 2025 Nov 28;16:10743. doi: 10.1038/s41467-025-65776-y (PMC12663454; doi:10.1038/s41467-025-65776-y)
Supplement: Supplementary file 1 — Supplementary Information [file 41467_2025_65776_MOESM1_ESM.pdf]

Supplementary Fig. 1

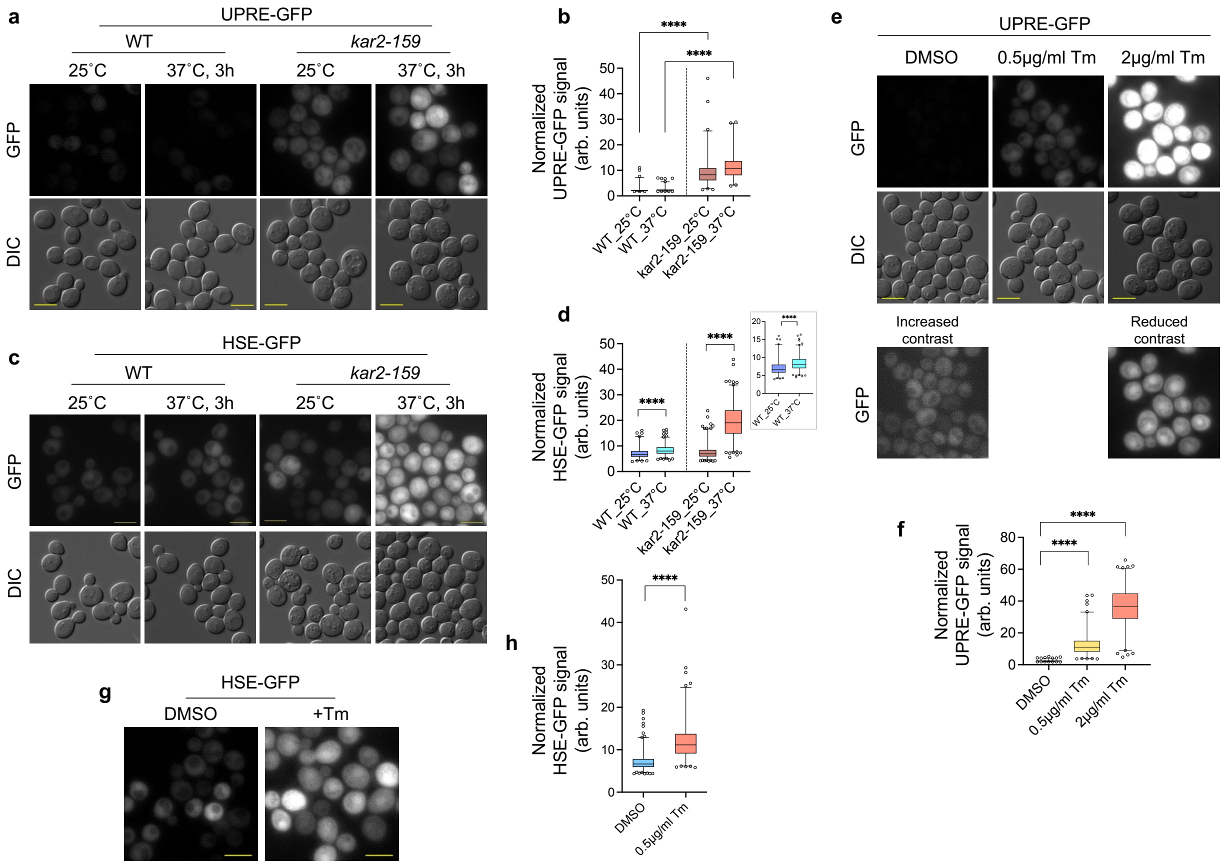

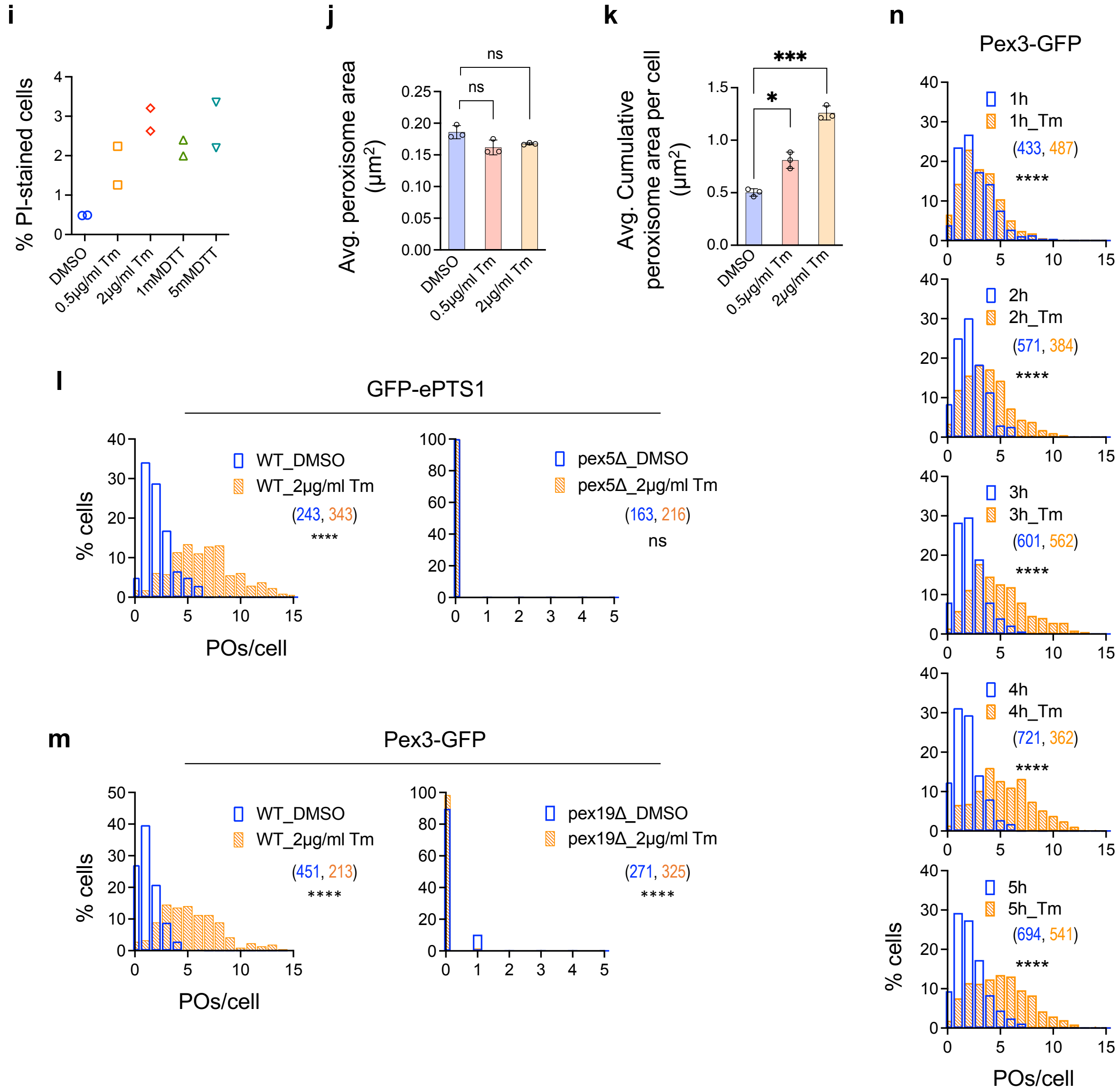

Supplementary Fig. 1: Protein misfolding induces UPR, HSR and peroxisomes.
a-d) Single Z-slice images (a, c) and quantification (b, d) showing UPRE-GFP (a-b) or HSE-GFP (c-d) levels per cell in WT and *kar2-159* cells after growth at 25°C and after 3h of growth at 37°C. Inset in (d) to show WT at different temperatures [(b) N_{cells} for WT: 25°C: 337, 37°C: 594; N_{cells} for *kar2-159*: 25°C: 362, 37°C: 231; (d) N_{cells} for WT (same data for WT as in Figure 3e): 25°C: 416, 37°C: 620; N_{cells} for *kar2-159*: 25°C: 863, 37°C: 795]. e-h) Single Z-slice images (e, g) and quantification (f, h) of UPRE-GFP and HSE-GFP levels in WT cells after treatment with either tunicamycin (Tm) or DMSO for 5h [(f) N_{cells}: DMSO: 786, 0.5µg/ml Tm: 531, 2µg/ml Tm: 532; (h) N_{cells}: DMSO: 819, 0.5µg/ml Tm: 546]. i) Effect of tunicamycin and DTT on cell death quantified by measuring the proportion of PI-stained cells at 5h after treatment with the indicated concentrations of the two ER stressors. j-k) average area of individual peroxisomes per cell (j) and average of the cumulative area occupied by all the peroxisomes per cell (k) quantified after 5h of treatment with DMSO or tunicamycin [n=3 experiments, N_{cells}: DMSO: 1120,

0.5μg/ml Tm: 1108; 2μg/ml Tm: 881; $N_{peroxisomes}$ DMSO: 3133, 0.5μg/ml Tm: 5714; 2μg/ml Tm: 6869; Mean ± SE for combined replicates: for (j), DMSO: 0.1859±0.0060, 0.5μg/ml Tm: 0.1618±0.0067, 2μg/ml Tm: 0.1675±0.0008; for (k): DMSO: 0.5021±0.0206, 0.5μg/ml Tm: 0.8088±0.0447, 2μg/ml Tm: 1.260±0.0383; Two-tailed Unpaired t test with Welch's correction; for (j), DMSO vs 0.5μg/ml Tm: P=0.0567, df=3.950, DMSO vs 2μg/ml Tm: P=0.0897, df=2.081; for (k), DMSO vs 0.5μg/ml Tm: P=0.0101, df=2.815, DMSO vs 2μg/ml Tm: P=0.0004, df=3.068]. l) Histograms showing number of GFP-ePTS1 puncta in WT and *pex5*Δ, at 5h after treatment with 2μg/ml tunicamycin; *pex5*Δ cells are unable to import GFP-ePTS1 into peroxisomes thereby serving as a control to account for possible non-peroxisomal GFP-ePTS1 puncta. m) Histograms showing the number of Pex3-GFP puncta in WT or *pex19*Δ cells after treatment with 2μg/ml tunicamycin; *pex19*Δ cells cannot make peroxisomes and hence serve as a control to account for possible occurrence of non-peroxisomal puncta. n) Histograms showing the number of Pex3-GFP marked peroxisomes at different timepoints after treatment with DMSO or 0.5μg/ml tunicamycin. Scale bar in all images: 5μm. In all histograms, N_{cells} indicated in parentheses; Two-tailed Mann-Whitney test: ****, P<0.0001. In all panels, comparison considered significantly different if P<0.05. In all box plots, bounds of the box: IQR, centre line: median, whiskers: 1-99 percentile, points: top and bottom 1 percentile; Two-tailed Mann-Whitney test: ****, P<0.0001.

Supplementary Fig. 2

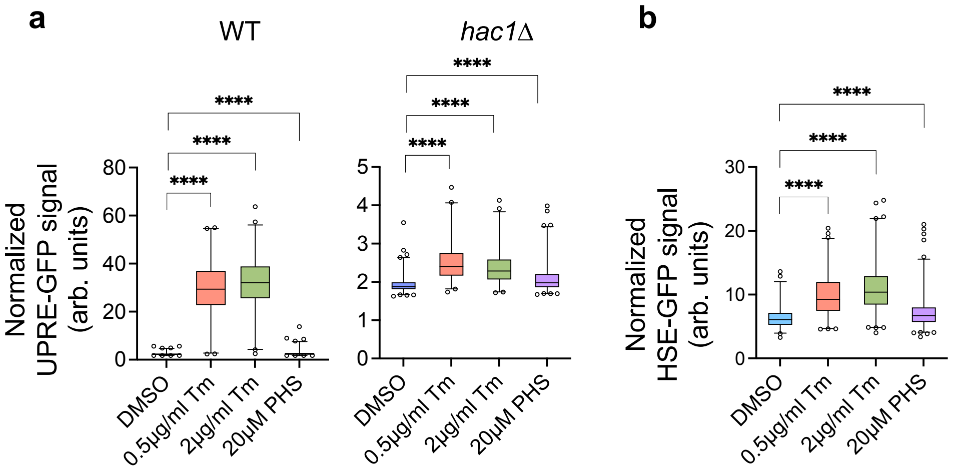

Supplementary Fig. 2: Effect of ERSU activation on UPR and HSR induction.
a-b) Comparison of UPRE-GFP (a) or HSE-GFP (b) levels at 5h after treatment with Phytosphingosine (PHS) or tunicamycin [(a) N_{cells} for WT: DMSO: 466, 0.5µg/ml Tm: 245, 2µg/ml Tm: 267, 20µM PHS: 424; N_{cells} for *hac1*Δ: DMSO: 440, 0.5µg/ml Tm: 262, 2µg/ml Tm: 298, 20µM PHS: 456; (b) N_{cells}: DMSO: 272, 0.5µg/ml Tm: 347, 2µg/ml Tm: 402, 20µM PHS: 553]. In all box plots, bounds of the box: IQR, centre line: median, whiskers: 1-99 percentile, points: top and bottom 1 percentile; Two-tailed Mann-Whitney test: ****, $P<0.0001$.

Supplementary Fig. 3

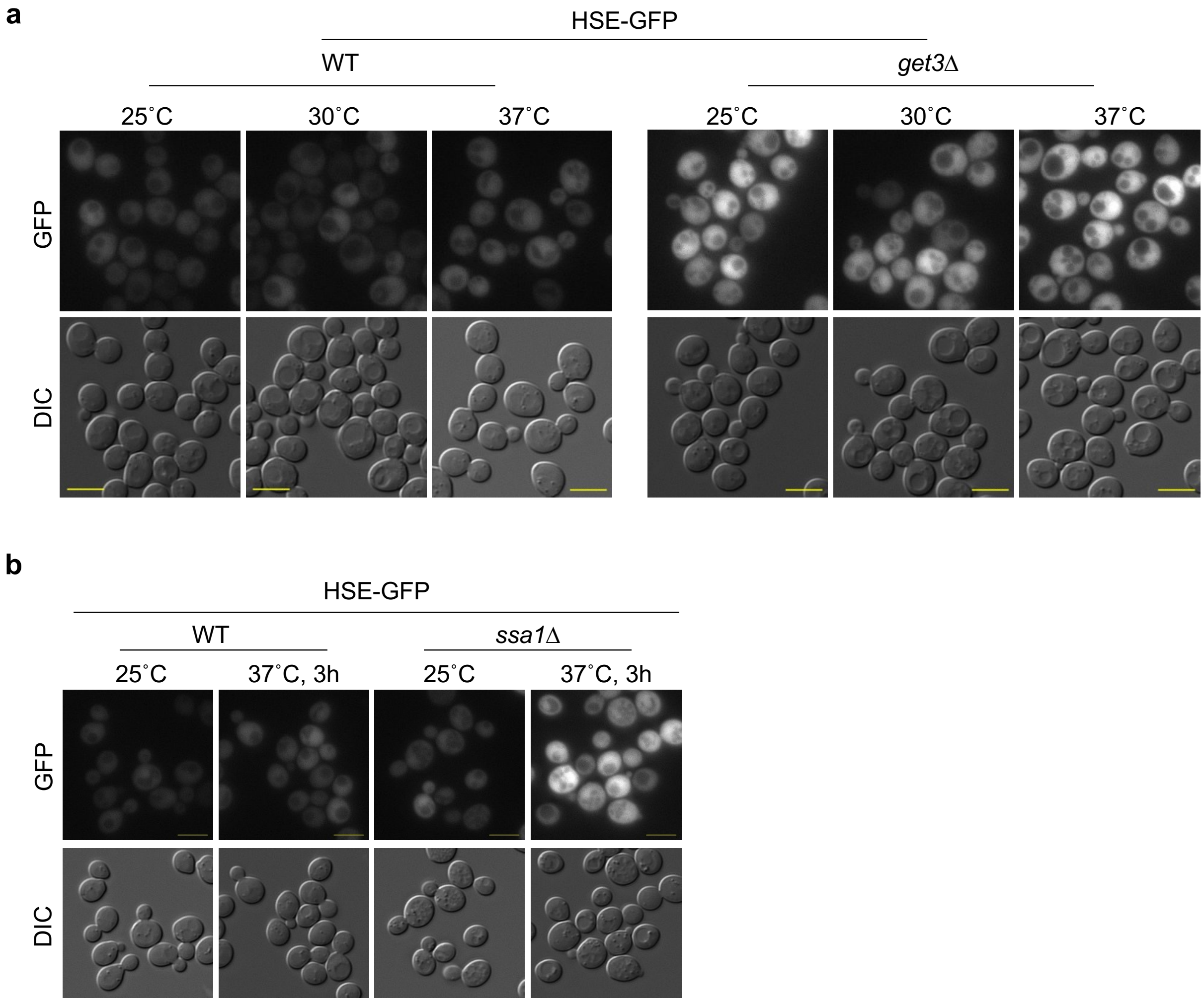

Supplementary Fig. 3: Loss of Get3 or Ssa1 activates HSR.
a-b) Single Z-slice images showing HSE-GFP signal increases in *get3*Δ and *ssa1*Δ cells. Scale bar: 5μm.

Supplementary Fig. 4

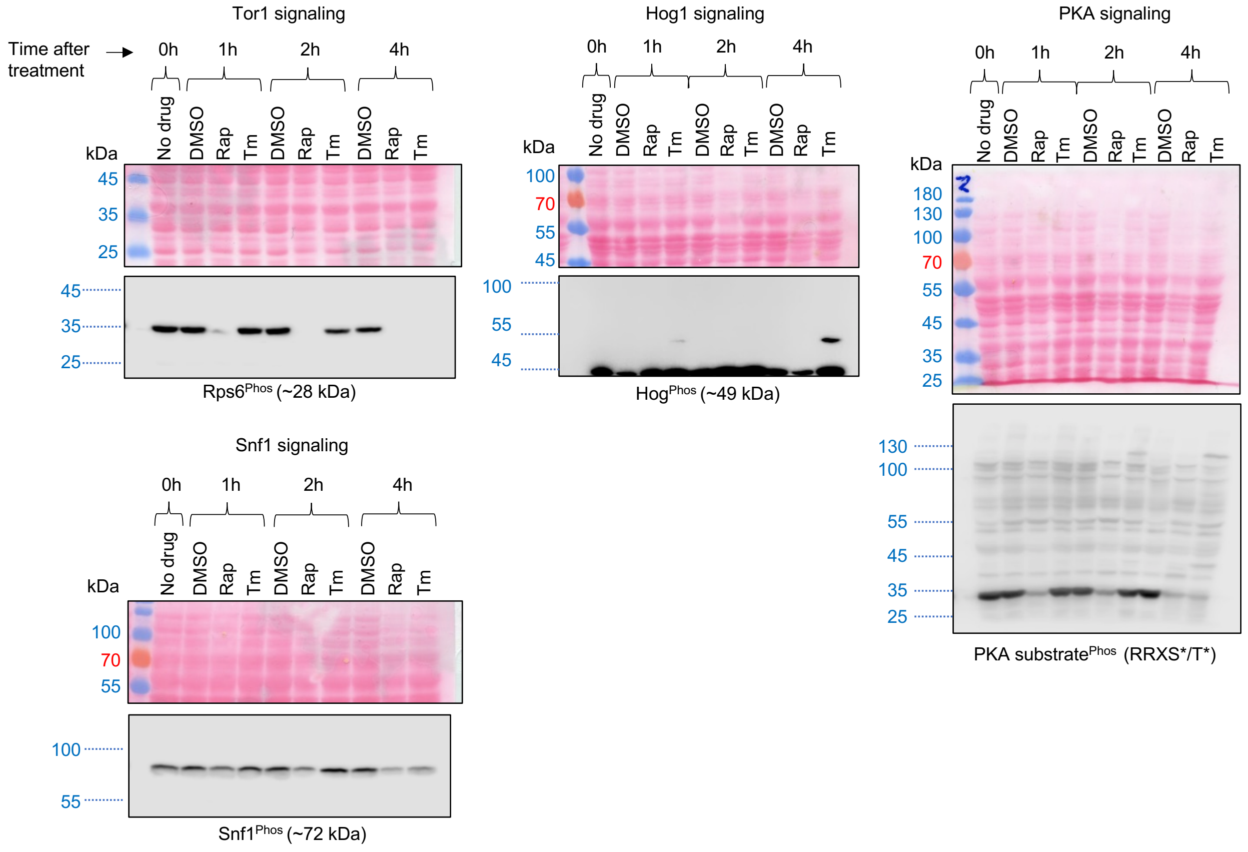

Supplementary Fig. 4: Effects of tunicamycin treatment on multiple signaling pathways. Western blots showing the levels of phosphorylated Rps6, Snf1, and Hog1 to visualize activation of TOR-, SNF1- and HOG- pathways, respectively, at different time points after 0.5µg/mL tunicamycin and 0.5µg/mL rapamycin treatments in comparison to DMSO control. Changes in PKA activity tested by probing for changes in phosphorylation profile of PKA substrate. Ponceau (top image for each blot) and actin (shown in Source Data) used as a loading controls. $Rps6^{Phos}$ and $Snf1^{\ Phos}$ were probed on the same nitrocellulose membrane whereas $Hog1^{Phos}$ was probed on a different membrane. PKA substrate ^{Phos} was also probed on a different membrane. All the three membranes (representing samples loaded on three different gels) were stained with Ponceau as well as for probed for actin. Uncropped blots along with merge images with the molecular weight marker are shown in Source Data (n=1).

Supplementary Fig. 5

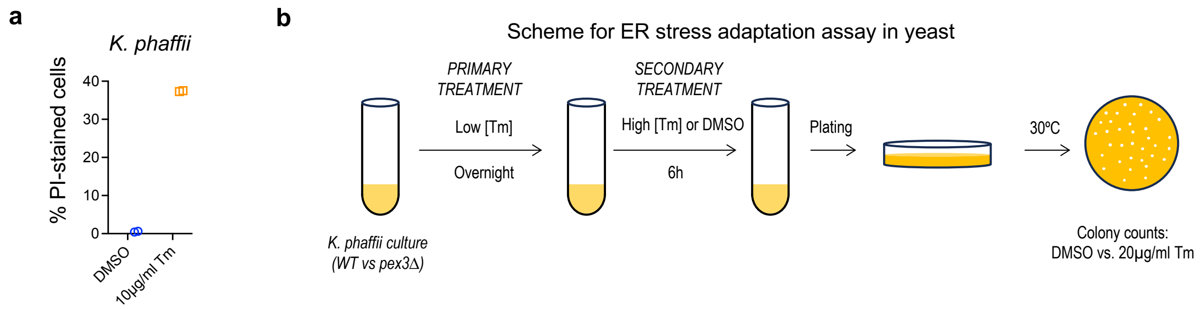

Supplementary Fig. 5: Effects of tunicamycin on cell viability in *K. phaffii*.
a) Effect of 10µg/ml tunicamycin treatment on the survival of *K. phaffii* cells measured using PI staining. b) Scheme for quantifying the adaptation of WT and *pex3*Δ *K. phaffii* cells to ER stress.

Supplementary Fig. 6

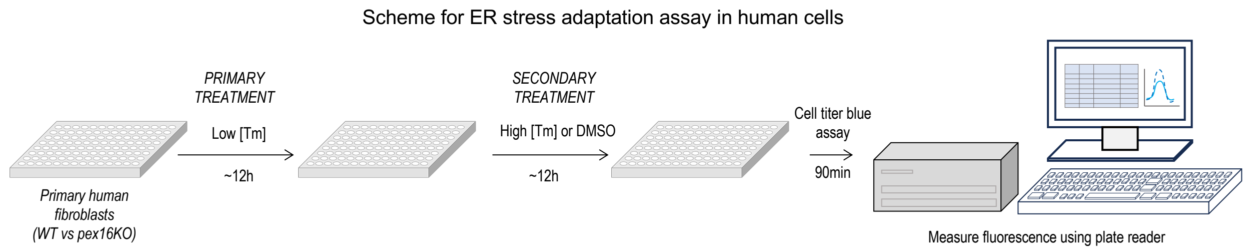

Supplementary Fig. 6: Effects of tunicamycin on cell viability in human fibroblasts. The schematic demonstrates the experimental format for testing for ER stress adaptation in human primary fibroblasts. Cells were seeded in 384 well plates and treated with a titration of tunicamycin (Tm; 80ng/ml, 155ng/ml, 310ng/ml, 625ng/ml, 1.2µg/ml, 2.5µg/ml, 5.0µg/ml, 10µg/ml and 20µg/ml) or DMSO with treatment assigned randomly across the plate. After 12h a secondary treatment of Tm (20µg/ml) or DMSO was overlayed on the wells. After a 12h incubation, viability was measured using CellTiter-Blue (Promega).

Supplementary Fig. 7

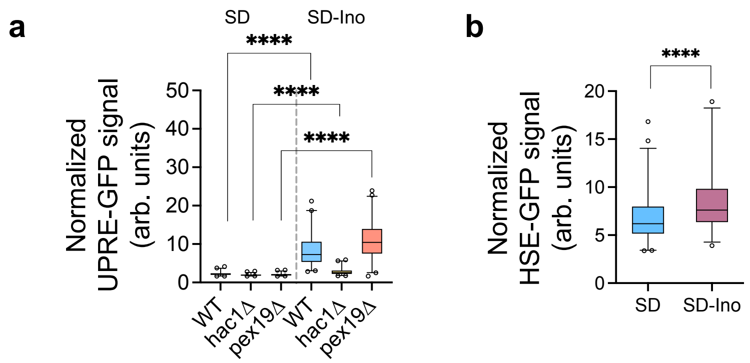

Supplementary Fig. 7: Effect of inositol deprivation on UPR and HSR activation.
a) UPRE-GFP levels per cell in WT, *hac1*Δ and *pex19*Δ growing in SD media with or without inositol (N_{cells}: SD: WT: 245, *hac1*Δ: 270, *pex19*Δ: 263; SD-Ino: WT: 234, *hac1*Δ: 241, *pex19*Δ: 236). b) Levels of HSE-GFP per cell in WT grown in SD media with or without Inositol (N_{cells}: SD: 291, SD-Ino: 172). In both plots, bounds of the box: IQR, centre line: median, whiskers: 1-99 percentile, points: top and bottom 1 percentile; Two-tailed Mann-Whitney test: ****, P<0.0001.

Abbreviations
CSM: Complete Supplement Mixture
ER: endoplasmic reticulum
DIC: Differential Interference Contrast
GET: Guided entry of tail-anchored proteins
GFP: Green Fluorescent Protein
HSR: Heat Shock Response
HSE: Heat Shock Response Element
K. phaffii: *Komagataella phaffii*
PMP: Peroxisomal membrane protein
PO: peroxisome
PTS: peroxisomal targeting signal
S. cerevisiae: *Saccharomyces cerevisiae*
SD: synthetic defined
STE: steryl esters
TAG: triacylglycerol
ts: temperature sensitive
YE: Yeast extract
YNB: Yeast nitrogen base
UPR: Unfolded Protein Response
UPRE: Unfolded Protein Response Element
WT: Wild type (n)/ wild-type (adj.)

References for Supplementary Data 1:

71. Mast, F. D. *et al.* Peroxins Pex30 and Pex29 Dynamically Associate with Reticulons to Regulate Peroxisome Biogenesis from the Endoplasmic Reticulum. *Journal of Biological Chemistry* 291, 15408–15427 (2016).
72. DeLoache, W. C., Russ, Z. N. & Dueber, J. E. Towards repurposing the yeast peroxisome for compartmentalizing heterologous metabolic pathways. *Nat Commun* 7, 11152 (2016).
73. Li, Z. *et al.* Systematic exploration of essential yeast gene function with temperature-sensitive mutants. *Nat Biotechnol* 29, 361–367 (2011).
74. Takahara, T. & Maeda, T. Transient Sequestration of TORC1 into Stress Granules during Heat Stress. *Mol Cell* 47, 242–252 (2012).
75. Winzeler, E. A. *et al.* Functional Characterization of the *S. cerevisiae* Genome by Gene Deletion and Parallel Analysis. *Science (1979)* 285, 901–906 (1999).
