# Supplementary material for: TOR and heat shock response pathways regulate peroxisome biogenesis during proteotoxic stress
Source: Nat Commun. 2025 Nov 28;16:10743. doi: 10.1038/s41467-025-65776-y (PMC12663454; doi:10.1038/s41467-025-65776-y)
Supplement: Supplementary file 2 — Description of Additional Supplementary Files [file 41467_2025_65776_MOESM2_ESM.pdf]

Description of Additional Supplementary Files

Supplementary Data 1: Microsoft Excel file containing a list of strains used in this study.

Supplementary Data 2: Microsoft Excel file containing data organized as experimental batches and strain construction details.
