# Supplementary material for: TOR and heat shock response pathways regulate peroxisome biogenesis during proteotoxic stress
Source: Nat Commun. 2025 Nov 28;16:10743. doi: 10.1038/s41467-025-65776-y (PMC12663454; doi:10.1038/s41467-025-65776-y)
Supplement: Supplementary file 5 — Reporting Summary [file 41467_2025_65776_MOESM5_ESM.pdf]

nature portfolio

Corresponding author(s): Suresh Subramani, Nandini Shukla

Last updated by author(s): 09/30/2025

Reporting Summary

Nature Portfolio wishes to improve the reproducibility of the work that we publish. This form provides structure for consistency and transparency in reporting. For further information on Nature Portfolio policies, see our Editorial Policies and the Editorial Policy Checklist.

Statistics

For all statistical analyses, confirm that the following items are present in the figure legend, table legend, main text, or Methods section.

n/a	Confirmed	
☐	☒	The exact sample size (*n*) for each experimental group/condition, given as a discrete number and unit of measurement
☐	☒	A statement on whether measurements were taken from distinct samples or whether the same sample was measured repeatedly
☐	☒	The statistical test(s) used AND whether they are one- or two-sided<br>*Only common tests should be described solely by name; describe more complex techniques in the Methods section.*
☒	☐	A description of all covariates tested
☐	☒	A description of any assumptions or corrections, such as tests of normality and adjustment for multiple comparisons
☐	☒	A full description of the statistical parameters including central tendency (e.g. means) or other basic estimates (e.g. regression coefficient) AND variation (e.g. standard deviation) or associated estimates of uncertainty (e.g. confidence intervals)
☐	☒	For null hypothesis testing, the test statistic (e.g. *F*, *t*, *r*) with confidence intervals, effect sizes, degrees of freedom and *P* value noted<br>*Give P values as exact values whenever suitable.*
☒	☐	For Bayesian analysis, information on the choice of priors and Markov chain Monte Carlo settings
☒	☐	For hierarchical and complex designs, identification of the appropriate level for tests and full reporting of outcomes
☒	☐	Estimates of effect sizes (e.g. Cohen's *d*, Pearson's *r*), indicating how they were calculated

Our web collection on statistics for biologists contains articles on many of the points above.

Software and code

Policy information about availability of computer code

Data collection	Not applicable
Data analysis	GraphPad Prism V10.4.0, FIJI/ImageJ2 V2.14.0; R version 4.4.0; pscl" R package (version 1.5.9), perox-per-cell V0.0.6 and code for image processing of human cells (V 0.1.0-NCOMMS) available on github (link in Code Availability).

For manuscripts utilizing custom algorithms or software that are central to the research but not yet described in published literature, software must be made available to editors and reviewers. We strongly encourage code deposition in a community repository (e.g. GitHub). See the Nature Portfolio guidelines for submitting code & software for further information.

Data

Policy information about availability of data

All manuscripts must include a data availability statement. This statement should provide the following information, where applicable:

- Accession codes, unique identifiers, or web links for publicly available datasets
- A description of any restrictions on data availability
- For clinical datasets or third party data, please ensure that the statement adheres to our policy

All data generated in this study are provided in the Source Data/Supplementary Data files.

Research involving human participants, their data, or biological material

Policy information about studies with human participants or human data. See also policy information about sex, gender (identity/presentation), and sexual orientation and race, ethnicity and racism.

Reporting on sex and gender	Not applicable
Reporting on race, ethnicity, or other socially relevant groupings	Not applicable
Population characteristics	Not applicable
Recruitment	Not applicable
Ethics oversight	Not applicable

Note that full information on the approval of the study protocol must also be provided in the manuscript.

Field-specific reporting

Please select the one below that is the best fit for your research. If you are not sure, read the appropriate sections before making your selection.

☒ Life sciences ☐ Behavioural & social sciences ☐ Ecological, evolutionary & environmental sciences

For a reference copy of the document with all sections, see nature.com/documents/nr-reporting-summary-flat.pdf

Life sciences study design

All studies must disclose on these points even when the disclosure is negative.

Sample size	No formal sample size calculation was performed. Sample sizes were chosen to balance feasibility with sufficient power to detect consistent patterns across replicates. Our sample sizes exceed similar imaging-based assays on peroxisome number.
Data exclusions	Visibly dead/severely damaged cells were manually excluded from downstream analyses, graphing and subsequent conclusions for kar2-159 and hac1Δ, and their corresponding WT control as explained in the methods section. Dead cells were not excluded for GLM analyses. Data from all cells is shown in Source Data/Supplementary Data 2.
Replication	Replication attempts for all experiments were successful. Where variability was observed, additional replicates were performed to increase statistical power and improve the precision of the results. Data from all independent replications is in Source Data/Supplementary Data 2.
Randomization	No random allocation was performed. Experiments were designed using established yeast genetics, where defined mutant strains were compared directly to wild-type controls. Mutants were grouped within the same experiment based on comparable growth characteristics, pathway membership, or related biological functions, ensuring that comparisons were biologically meaningful. Importantly, batch and plate effects were controlled in our GLM analyses.
Blinding	Data collection was not blinded because pre-determined mutants or treatments were examined to test their respective hypotheses.

Reporting for specific materials, systems and methods

We require information from authors about some types of materials, experimental systems and methods used in many studies. Here, indicate whether each material, system or method listed is relevant to your study. If you are not sure if a list item applies to your research, read the appropriate section before selecting a response.

Materials & experimental systems

n/a	Involved in the study	
☐	☒	Antibodies
☒	☐	Eukaryotic cell lines
☒	☐	Palaeontology and archaeology
☒	☐	Animals and other organisms
☒	☐	Clinical data
☒	☐	Dual use research of concern
☒	☐	Plants

Methods

n/a	Involved in the study	
☒	☐	ChIP-seq
☒	☐	Flow cytometry
☒	☐	MRI-based neuroimaging

Antibodies

Antibodies used	Phospho-S6 Ribosomal Protein (Ser235/236) (D57.2.2E) XP Rabbit mAb, Cell Signaling Technology (#4858, 1:2000); Phospho-AMPKα (Thr172) (40H9) Rabbit mAb, Cell Signaling Technology (#2535, 1:2000); Phospho-p38 MAPK (Thr180/Tyr182) (D3F9) XP Rabbit mAb, Cell Signaling Technology (#4511, 1:2000); Phospho-PKA Substrate (RRXS*/T*) (100G7E) Rabbit mAb, Cell Signaling Technology (#9624, 1:2000); Anti-ScActin Rabbit pAb20, Gift from Michael Yaffe (1:5000); Goat Anti-Rabbit IgG (H+L)-HRP Conjugate, Bio-RAD; (#1721019, 1:5000); anti-PMP70 antibody, Abcam (EPR5614; 1:1000); donkey anti-Rabbit IgG (H+L)-Alexa Fluor 488, Thermo Fisher Scientific (A-21206, 1:2000)
Validation	All antibodies have been used before in other published studies; information for commercial sources or references is provided.

Plants

Seed stocks	*Report on the source of all seed stocks or other plant material used. If applicable, state the seed stock centre and catalogue number. If plant specimens were collected from the field, describe the collection location, date and sampling procedures.*
Novel plant genotypes	*Describe the methods by which all novel plant genotypes were produced. This includes those generated by transgenic approaches, gene editing, chemical/radiation-based mutagenesis and hybridization. For transgenic lines, describe the transformation method, the number of independent lines analyzed and the generation upon which experiments were performed. For gene-edited lines, describe the editor used, the endogenous sequence targeted for editing, the targeting guide RNA sequence (if applicable) and how the editor was applied.*
Authentication	*Describe any authentication procedures for each seed stock used or novel genotype generated. Describe any experiments used to assess the effect of a mutation and, where applicable, how potential secondary effects (e.g. second site T-DNA insertions, mosiacism, off-target gene editing) were examined.*
